# Penetration of linezolid into bone tissue 24 h after administration in patients with multidrug-resistant spinal tuberculosis

**DOI:** 10.1371/journal.pone.0223391

**Published:** 2019-10-03

**Authors:** Yuan Li, Hairong Huang, Weijie Dong, Tinglong Lan, Jun Fan, Shu’an Wen, Tingting Zhang, Shibing Qin, Ai Guo

**Affiliations:** 1 Department of Orthopedics, Beijing Friendship Hospital, Capital Medical University, Beijing, China; 2 Department of Orthopedics, Beijing Chest Hospital, Capital Medical University, Beijing, China; 3 National Clinical Laboratory on Tuberculosis, Beijing Key Laboratory for Drug Resistant Tuberculosis Research, Beijing Chest Hospital, Capital Medical University, Beijing Tuberculosis and Thoracic Tumor Institute, Beijing, China; University of Groningen, University Medical Center Groningen, NETHERLANDS

## Abstract

**Background:**

Linezolid has shown strong antimicrobial activity against multidrug-resistant (MDR)/rifampin-resistant strains of *Mycobacterium tuberculosis*. Linezolid achieves clinical efficacy mainly through area under the concentration time curve/minimum inhibitory concentration ratio in the infected lesion site. Previous studies mainly focused on the relationship between linezolid concentrations in the blood and infected bone tissue when the blood drug concentration reached the peak 2 h after administration. However, we do not know whether linezolid can maintain the same bone/plasma ratio in infected bone tissue when the blood concentration reaches the trough level. Therefore, this study aimed to evaluate the penetrability of linezolid into bone tissue 24 h after administration in patients with MDR spinal tuberculosis (TB).

**Methods:**

Nine MDR spinal TB patients, who received a treatment regimen including linezolid and underwent surgery, were enrolled prospectively from April 2017 to March 2019. Blood and diseased bone tissue specimens were collected simultaneously during operations 24 h after taking 600 mg of linezolid orally. Linezolid concentrations in plasma and diseased bone tissue specimens were determined by high-performance liquid chromatography–tandem mass spectrometry.

**Results:**

Following a 600 mg oral administration of linezolid 24 h before surgery, median concentrations of linezolid in plasma and diseased bone tissue for the 9 patients were 1.98 mg/L (range 0.30–3.44 mg/L) and 0.60 mg/L (range 0.18–2.13 mg/L), respectively, at resection time. The median diseased bone/plasma linezolid concentration ratio was 0.48 (range 0.30–0.67). Pearson’s correlation analysis showed that linezolid concentrations in the plasma were positively related to those in diseased bone tissue (r = 0.949, p < 0.001).

**Conclusions:**

After 24 h of medication, linezolid still had good penetrability into diseased bone tissue in patients with MDR spinal TB.

## Introduction

Tuberculosis (TB) is still one of the top 10 causes of death, and the leading cause from a single infectious agent worldwide [1]. Bone and joint TB accounts for 1%–3% of all TB cases. Globally, 3.5% of new TB cases and 18% of previously treated cases were multidrug-resistant (MDR)/rifampicin-resistant (RR)-TB, and the treatment success rate for MDR/RR-TB cases started on second-line treatment regimen is only 41% in China [1].

Linezolid has demonstrated potent activity against MDR and extensively drug-resistant *Mycobacterium tuberculosis* [2–5]. Linezolid has become an important component of treatment regimens for MDR-TB and is classified as a group A drug in the latest World Health Organization (WHO) treatment guidelines for MDR-TB and RR-TB [6]. Previous studies showed that Linezolid achieved clinical efficacy against *Mycobacterium tuberculosis* mainly through area under the concentration time curve (AUC)/minimum inhibitory concentration (MIC) ratio [4,7]. But previous studies on pharmacokinetics/pharmacodynamics of linezolid mainly used blood samples, so we do not know the drug concentration of linezolid in the lesions.

Previous studies found that linezolid could effectively penetrate into infected bone tissue when the blood drug concentration reached the peak 2 h after administration [8–10]. However, we do not know whether linezolid can maintain the same bone/plasma ratio in infected bone tissue when the blood concentration reaches the trough level. Therefore, this study aimed to evaluate the penetrability of linezolid into bone tissue 24 h after administration in patients with MDR spinal TB, which could give us a more comprehensive understanding of the distribution of linezloid in bone lesions at different time points.

## Materials and methods

### Ethics statement

This study was established in accordance with the ethical guidelines of the Helsinki Declaration and was approved by the Human Ethics Committee of Beijing Chest Hospital, Capital Medical University. Written informed consent was obtained from individual participants.

### Patient categories

From April 2017 to March 2019, patients with culture-confirmed MDR spinal TB were enrolled prospectively in the Orthopedics Department of Beijing Chest Hospital, Capital Medical University. They received treatment regimen including linezolid and underwent adjunctive surgical resection. Indications for adjunctive surgery included: 1) progressive bone destruction led to instability of the spine, and no improvement was observed after anti-tuberculosis treatment; 2) progressive neurological dysfunction, and no improvement was observed after anti-tuberculosis treatment. The patients were aged between 18 years and 72 years.

Patients were excluded if they met the following exclusion criteria: 1) allergic to linezolid; 2) severe cardiovascular, liver, kidney or blood system disease or other serious illnesses; 3) mentally ill; 4) pregnant or lactating females; 5) a positive HIV test result.

### Study design

Patients with suspected MDR spinal TB were enrolled prospectively. Then, they were diagnosed with MDR spinal TB by computed tomography-guided puncture biopsy and TB culture (BACTEC MGIT 960) and drug susceptibility test (DST). Patients were given individually based chemotherapy regimens on the basis of medication history and DST results. Each regimen consisted of five drugs, including linezolid. The drugs were selected in accordance with the WHO treatment guidelines for drug-resistant TB [11]. All patients took linezolid at a dose of 600 mg per day orally. The patients received anti-TB treatment for 4–8 weeks before surgery. Then, they underwent surgical resection. Patients received 600 mg of linezolid orally 24 h before surgery. Blood and diseased bone tissue specimens were collected simultaneously during operations. Linezolid concentrations in plasma and diseased bone tissue specimens were determined by high-performance liquid chromatography–tandem mass spectrometry (HPLC-MS/MS).

### Sampling

We selected diseased bone tissue samples from spinal TB lesions, which had been infected with *Mycobacterium tuberculosis* but were still alive, not sequestrum. Each specimen included cancellous and cortical bone. Venous blood and diseased bone tissue samples were collected simultaneously during operations. Samples were obtained 24 h after drug oral ingestion. Plasma was separated using centrifugation at 3000 × g for 15 min. Both plasma and diseased bone tissue samples were stored at −80°C until analysis. All standard stock solutions were prepared at 1 mg/mL. Linezolid and its internal standard (IS) zaleplon were dissolved in methanol and stored at −70 °C prior to use.

Briefly, after weighing the diseased bone tissue sample, one volume of ultrapure water was added, and all diseased bone tissue samples were homogenized in a FastPrep-24 Instrument (MP Biomedicals Europe) for 100 s at 6 m/s by MP Bio FASTPREP-24. Homogenates were placed at 4°C for 5 h, vortexing every 30 min, for drug extraction. After centrifugation at 3500×g for 15 min, the supernatant was separated. Methanol and IS were added into the supernatant separated from diseased bone tissue homogenates or plasma separated from blood samples and mixed thoroughly. After centrifugation for a further 15 min, the supernatant was transferred to glass injection vials for HPLC analysis.

### HPLC-MS/MS analysis

Linezolid concentrations in plasma and diseased bone tissue were analyzed using a validated HPLC-MS/MS method with an HPLC systems (Agilent Technologies, Santa Clara, CA, USA) equipped with an autosampler (G1329A) and a column heater (G1316A). We used a G6420A triple quadrupole mass spectrometer (Agilent Technologies) and an electrospray ionization source. The mass spectrometer was operated using the following settings: 5 kV capillary voltage and 22–27 eV collision energy. Quantification was achieved by selected reaction monitoring (SRM) in positive ion mode. Integration of peak area and data analysis were both performed using Agilent MassHunter software B.08.00 (Agilent Technologies, Santa Clara, CA, USA). Analysis for linezolid was performed using a ZORBAX SB-C18 column (2.1 × 50 mm; Agilent Technologies).

The mobile phase comprised acetonitrile and 0.1% formic acid solution (65:35, v/v) at a flow rate of 0.3 mL/min. Multiple reaction monitoring transition was 338.1→296.1 for linezolid. Calibration curves in the range of 0.2–25 μg/mL for linezolid were established (r^2^ = 0.9989).

### Data management and statistical analysis

All data were entered into a Microsoft Office Excel file. The continuous variables in this study were presented as the median. The relationships between plasma and diseased bone tissue concentrations of linezolid were examined using Pearson’s correlation. All tests of significance were two-tailed, and p < 0.05 was considered statistically significant. Analysis was performed using the commercial statistical software SPSS version 13.0 (SPSS Inc, Chicago, IL, USA).

## Results

### Study patients

Nine patients (four males and five females) with a median age of 50 years were enrolled. The median body mass index (BMI) was 21.48 kg·m^−2^ (range 17.30–27.34 kg·m^−2^). All patients were diagnosed with MDR spinal TB and underwent operation. Patients received linezolid for an average duration of 40 ± 6 days before operation. All patients were administered with daily doses of linezolid (600 mg), and the median linezolid dose by weight was 10.53 mg·kg^−1^ (range 7.59–12.00 mg·kg^−1^). Table 1 shows clinical characteristics of 9 studied patients.

**Table 1:** Clinical characteristics of 9 studied patients.

Patient serial No.	Gender	Age (years)	Lesion site	BMI (Kg/m^2^)	Dose/Weight (mg/kg)	Duration of anti-tuberculosis treatment before surgery (days)	Anti-tuberculosis treatment regimen
1	male	49	T11-12	24.22	9.68	45	Z-Lfx-Lzd-Am-PAS
2	male	65	T10-11	25.50	7.59	33	Z-Lfx-Lzd-Am-Pto
3	female	69	T4-5	21.48	10.91	49	Z-Mfx-Lzd-Am-Pto
4	male	69	T10-11	17.30	12.00	39	Z-Lfx-Lzd-Am-PAS
5	female	72	L4-5	27.34	8.57	40	Z-Mfx-Lzd-Am-PAS
6	male	18	C7-T3	17.30	12.00	42	Z-Lfx-Lzd-Am-Cs
7	female	27	L2-3	20.96	10.91	31	Z-Mfx-Lzd-Am-Pto
8	male	18	T12-L1	18.19	10.53	36	Z-Lfx-Lzd-Am-Cs
9	female	50	T9-11	24.43	9.84	46	Z-Lfx-Lzd-Am-Pto

No: number; T: thoracic vertebra; C: cervical vertebra; L: lumbar vertebra; BMI: body mass index; Dose/Weight: daily doses of linezolid (600 mg)/ patient’s weight; Z: pyrazinamide; Mfx: moxifloxacin; Lzd: linezolid; Am: amikacin; Pto: prothionamide; Cs: cycloserine; PAS: *p*-aminosalicylic acid.

### Linezolid concentrations in diseased bone and plasma

Following a 600 mg oral dose of linezolid 24 h before surgery, median plasma concentrations for the nine patients were 1.98 mg/L (range 0.30–3.44 mg/L) at resection time. With median diseased bone concentrations of 0.60 mg/L (range 0.18–2.13 mg/L), penetration of linezolid into diseased bone tissue was rapid. The median diseased bone/plasma linezolid concentration ratio was 0.48 (range 0.30–0.67). Linezolid concentrations in diseased bone tissue and plasma of all nine patients are shown in Table 2.

**Table 2:** Plasma and bone concentrations of linezolid 24 h after administration for each patient.

Patient serial No.	Linezolid concentrations in plasma (mg/L)	Linezolid concentrations in diseased bone tissue (mg/L)	Diseased bone/serum ratio
1	0.303	0.197	0.65
2	0.48	0.184	0.383
3	1.116	0.538	0.482
4	1.313	0.499	0.38
5	1.978	0.598	0.302
6	2.04	1.365	0.669
7	2.157	0.983	0.455
8	3.289	1.874	0.569
9	3.435	2.127	0.619
Median	1.98	0.60	0.48

### Correlation analysis of linezolid concentrations in diseased bone and plasma

**Fig 1:**
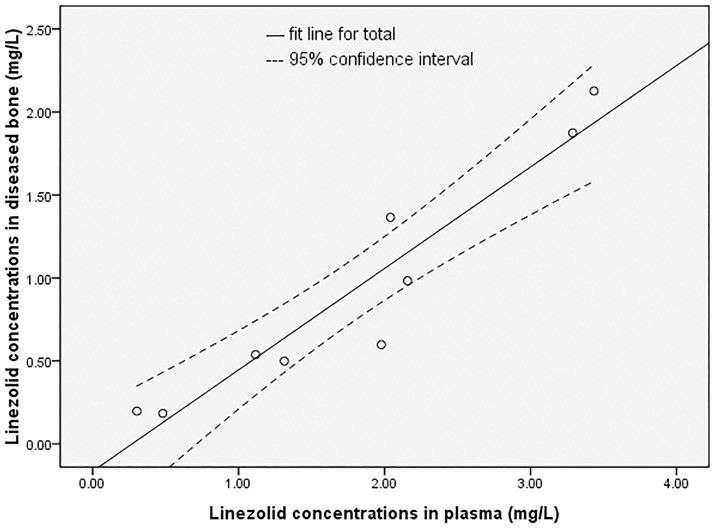
The scatter plot of linezolid concentrations in diseased bone and plasma of 9 patients.

Pearson’s correlation analysis showed that linezolid concentrations in plasma were positively related to those in diseased bone tissue (r = 0.949, p < 0.001). Fig 1 shows the scatter plot of linezolid concentrations in diseased bone and plasma. Pearson’s correlation analysis showed no correlation between diseased bone/plasma linezolid concentration ratio and linezolid plasma concentration, BMI, dose by weight (r = 0.246, p = 0.524; r = −0.325, p = 0.394; r = 0.367, p = 0.331).

## Discussion

At present, linezolid is widely used in the clinical treatment of MDR-TB. However, it has a high incidence of drug side effects associated with dosage and duration of MDR-TB treatment, leading to poor tolerance in patients [12–15]. So far, there is no consensus on the dosage of linezolid in MDR-TB treatment [16–17]. To optimize the dosage and administration method of linezolid, we need to understand its distribution in the lesion after patients take it.

Previous studies have evaluated the penetration of linezolid into bone tissue. However, most of them evaluated the penetration 2 h after drug administration, when the blood drug concentration peaked and previous studies did not cover patients with bone and joint TB [8–10]. The microdialysis method could only detect unbound extracellular concentrations of linezolid in specimens [18]. But *Mycobacterium tuberculosis* distributed simultaneously inside and outside the cell in diseased bone specimens. So we homogenized the diseased bone specimens, which released all the linezolid inside and outside the cell. We measured the overall concentration of linezolid in the lesion. The present study is the first prospective cohort study that investigated the penetrability of linezolid into bone tissue 24 h after administration in MDR spinal TB patients when the blood concentration reached the trough level. Determining the penetration ability of linezolid into bone tissue when blood drug concentration reaches a peak and trough is beneficial in guiding clinical dosages and administration method during the treatment of MDR spinal TB patients.

Twenty-four hours after taking 600 mg linezolid orally, the median concentrations of linezolid in diseased bone tissue were 0.6 mg/L, which was still higher than the suggested minimum inhibitory concentration cut-off of 0.5 mg·L^−1^. At the same time, linezolid concentrations in diseased bone tissue in 1/3 (3/9) of patients were above the generally accepted clinical susceptibility breakpoint of 1 mg·L^−1^ [19] and the mutant prevention concentration found in 90% of *M*. *tuberculosis* isolates in one study that used a concentration of 1.2 mg·L^−1^ [20]. The results of this study indicated that 5/9 (linezolid concentrations in diseased bone tissue >0.5mg·L^−1^) MDR spinal TB patients can achieve effective inhibitory concentration in diseased bone tissue when the blood concentration of linezolid reaches the trough concentration after taking 600mg of linezolid orally daily. This may be the reason why linezolid has achieved good results in the treatment of MDR spinal TB patients at 600 mg daily by mouth. Previous studies showed that AUC/MIC of 80–100 as a reasonable target to assure bactericidal activity against *Mycobacterium tuberculosis*, and limited pharmacokinetic studies in TB patients indicated that most, but not all, patients receiving linezolid 600 mg per day attained target plasma AUC/MIC values of ≥ 80–100 ug-h/ml, while most patients receiving 300 mg per day did not [21]. This also indicates that receiving linezolid 600 mg per day can achieve good anti-tuberculosis treatment effect. In this study, the variation of linezolid blood concentration may be due to individual differences in drug absorption, drug metabolism, BMI, and linezolid dose by weight. At present, no relevant research can explain the above hypotheses.

Previous studies have evaluated the penetration of linezolid into bone tissue 2 h after drug administration when the blood drug concentration reaches the peak concentration and reported bone/serum linezolid ratios of 0.2–0.6 [8–10]. In a study by Kempker et al, the median diseased lung tissue/serum linezolid concentration was 0.49 (range 0.18–0.92) among patients with drug-resistant TB [22]. In our study, the median bone/serum linezolid concentration ratio was 0.48 (range 0.30–0.67) 24 h after the administration of 600 mg linezolid, during which the blood drug concentration reached the trough level. Pearson’s correlation analysis showed that linezolid concentrations in plasma were positively related to those in diseased bone tissue. This result showed that linezolid still had good penetrability into diseased bone tissue after 24 h of administration. Referring to data reported by Rana et al, the penetrability of linezolid into diseased bone tissue was basically consistent [9], regardless of its peak or trough blood concentration. The positive correlation between linezolid concentrations in the bone and plasma made it possible to calculate the drug concentration in the bone by the blood concentration. Further regression analysis is required to establish the regression equation between the two variables. Pearson’s correlation analysis showed no correlation between diseased bone/plasma linezolid concentration ratio and linezolid plasma concentration, BMI and linezolid dose by weight. Variation of diseased bone/plasma linezolid concentration ratios may be caused by the variation of diseased bone structures. Further research is needed to clarify the above hypothesis.

Despite our findings, this study has several limitations. First, this study involved a small size cohort of patients with MDR spinal TB, which may not be representative of all patients with this disease. Second, we only measured linezolid concentrations in diseased bone tissue at one point in time, which may not represent the overall penetration of linezolid in bone lesions. Therefore, studies with large samples and measurement of linezolid concentrations at multiple time points are needed to further evaluate the penetrability of linezolid in bone lesions of patients with MDR spinal TB.

In summary, we found that linezolid had good penetrability into diseased bone tissue 24 h after administration in patients with MDR spinal TB, during which the blood drug concentration reached the trough level. This discovery may be helpful to optimize the dosage and administration method of linezolid in the treatment of patients with MDR spinal TB.
